# IL-6R Signaling Is Associated with PAD4 and Neutrophil Extracellular Trap Formation in Patients with STEMI

**DOI:** 10.3390/ijms26115348

**Published:** 2025-06-02

**Authors:** Kristine Mørk Kindberg, Jostein Nordeng, Miriam Sjåstad Langseth, Hossein Schandiz, Borghild Roald, Svein Solheim, Ingebjørg Seljeflot, Mathis Korseberg Stokke, Ragnhild Helseth

**Affiliations:** 1 Oslo Center for Clinical Heart Research, Department of Cardiology Ullevaal, Oslo University Hospital, 0450 Oslo, Norway; 2 Faculty of Medicine, Institute of Clinical Medicine, University of Oslo, 0450 Oslo, Norway; 3 Department of Cardiology Rikshospitalet, Oslo University Hospital, 0450 Oslo, Norway; 4 Department of Oncology, Akershus University Hospital, 1478 Lørenskog, Norway; 5 Department of Pathology, Oslo University Hospital Ullevaal, 0450 Oslo, Norway; 6 Institute for Experimental Medical Research, Oslo University Hospital and University of Oslo, 0450 Oslo, Norway

**Keywords:** coronary thrombus, NETs, STEMI, inflammation, IL-6R, PAD4

## Abstract

Inflammation contributes to myocardial injury in ST-elevation myocardial infarction (STEMI). Interleukin-6 receptor (IL-6R) inhibition has been shown to mitigate myocardial injury and reduce levels of the prothrombotic and inflammatory mediator, neutrophil extracellular traps (NETs). The enzyme peptidylarginine deiminase 4 (PAD4) is central in NET formation. We hypothesized that PAD4 links IL-6R activation and NET formation. Methods: We conducted thrombus aspiration and peripheral blood sampling in 33 STEMI patients. In thrombi and leukocytes, we quantified the mRNA of IL-6, IL-6R, and PAD4. In peripheral blood, the protein levels of IL-6, IL-6R, PAD4, dsDNA, H3Cit, MPO-DNA, and troponin T were quantified. Results: In thrombi and circulating leukocytes, PAD4 mRNA was associated with IL-6R mRNA (thrombi: β = 0.34, 95% CI [0.16–0.53], *p* = 0.001, circulating leukocytes: β = 0.92, 95% CI [0.07–1.77], *p* = 0.036). There were no correlations between PAD4 and IL-6 in thrombi and leukocytes. The protein levels of IL-6R were associated with the NET marker H3Cit (r_s_ = 0.40, *p* = 0.02). In thrombi, PAD4 mRNA was associated with high levels of troponin T (β = 1.15 95% CI [0.27–2.04], *p* = 0.013). Conclusion: We demonstrate an association between PAD4, IL-6R, and troponin release in STEMI patients. Our findings indicate a PAD4-mediated connection between IL-6R and NET formation and highlight PAD4 as a potential treatment target for mitigating inflammation and myocardial injury in STEMI.

## 1. Introduction

Circulating levels of interleukin (IL)-6 increase during the acute phase of ST-elevation myocardial infarction (STEMI) and are associated with infarct size and adverse clinical outcomes [1,2]. A central source of IL-6, which binds to the IL-6 receptor (IL-6R), is the NOD-, LRR-, and pyrin domain-containing protein 3 (NLRP3) inflammasome axis, a key mediator of inflammation [3]. A randomized, placebo-controlled trial, ASSessing the effect of Anti-IL-6 treatment in Myocardial Infarction (ASSAIL-MI), demonstrated that IL-6R inhibition with tocilizumab administered during PCI in patients with STEMI reduced the inflammatory response and myocardial damage [4]. Activation of IL-6R can mediate a vast array of both pro- and anti-inflammatory effects in cardiovascular diseases [5]. We recently reported that tocilizumab attenuates the formation of neutrophil extracellular traps (NETs) in the ASSAIL-MI trial and suggested that this could explain part of the IL-6R-mediated cardioprotective effect of tocilizumab [6].

NETs are extracellular web-like structures composed of double-stranded DNA (dsDNA), citrullinated histones, and various neutrophil proteins. They are released from activated neutrophils during STEMI in a process called NETosis [7,8]. NETs contribute to atherothrombosis through their clot-stabilizing, cytotoxic, and proinflammatory properties [9,10,11,12]. Circulating NET markers have repeatedly been linked to myocardial damage and clinical outcomes in STEMI [10,13], and NETs have been observed in coronary thrombi [14,15,16,17,18]. During NETosis, chromatin decondensation is initiated by the intracellular protein peptidylarginine deiminase 4 (PAD4) [19]. Inhibition of PAD4 has shown cardioprotective effects in preclinical models of myocardial infarction, making it a promising target for further studies [20]. We have previously identified an association between increased IL-6R gene expression and markers of myocardial damage in aspirated coronary thrombi from patients with STEMI [21]. In the present study, we hypothesized that IL-6R and NETosis are linked by PAD4. To test this, we analyzed aspirated coronary thrombi and circulating blood from patients with STEMI and assessed the associations between IL-6R, IL-6, PAD4, and NETs.

## 2. Results

### 2.1. Baseline Characteristics

The baseline characteristics of the study cohort are outlined in Table 1. The mean age was 60 years, and the female inclusion rate was 9%. The majority (79%) presented with total coronary occlusion prior to PCI, defined as TIMI flow 0. Twelve patients (36%) had retrograde flow to the culprit coronary artery, suggesting longstanding atherosclerotic disease. The available markers with their respective locations are listed in Table 2.

### 2.2. Investigations in Coronary Thrombi

#### 2.2.1. Associations Between PAD4, IL-6R, and IL-6 mRNA Levels

**Figure 1:**
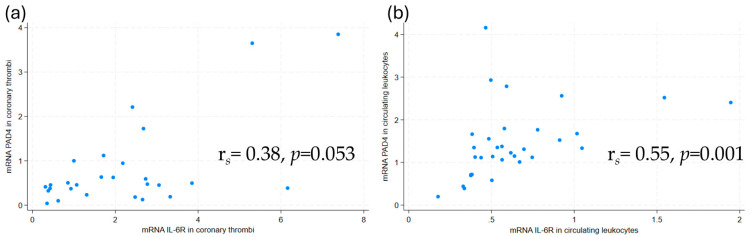
Scatterplots with Spearman’s Rho of PAD4 mRNA and IL-6R mRNA levels in (**a**) coronary thrombi and (**b**) circulating leukocytes. mRNA levels were measured by qPCR. PAD4 = peptidylarginine deiminase 4, IL-6R = interleukin-6 receptor.

PAD4 mRNA was detected in 27 of the 33 coronary thrombi. A correlation between mRNA for PAD4 and IL-6R in coronary thrombi was strongly suggested, although not statistically significant (Figure 1a). This association persisted, now statistically significant, in a multivariate, linear regression model adjusted for age, sex, and symptom duration (Table 3). In contrast, no association was found between PAD4 and IL-6 mRNA.

#### 2.2.2. Associations Between PAD4 mRNA Levels and Troponin T

The level of PAD4 mRNA in coronary thrombi was associated with high peak troponin T in a multivariate, linear regression model adjusted for age, sex, and symptom duration (Table 3). In contrast, PAD4 mRNA levels in circulating leukocytes, PAD4 protein levels, and the other circulating NET markers (dsDNA, MPO-DNA, and H3Cit) were not associated with troponin T.

#### 2.2.3. Immunofluorescence Investigations of NETosis

**Figure 2:**
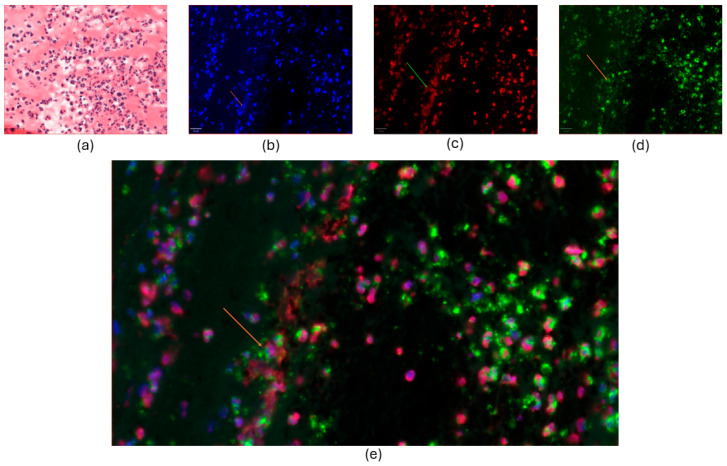
Co-localization of NET markers in coronary thrombi. (**a**): The HE-stained image displayed a cluster of abundant inflammatory cells, predominantly neutrophil granulocytes, with fibrin appearing as eosinophilic and amorphous deposit filaments. b-e: Immunofluorescence-stained thrombus, as in (**a**). (**b**): The nuclei and extracellular DNA were stained with DAPI (blue). (**c**): The nuclei were stained with histone H3 (red). On the left, web-like structures show extracellular deposits of histone H3. (**d**): Green-stained neutrophil elastase (NE) was abundant in the cytoplasm, cell membrane, and extracellular granular debris. (**e**): Co-staining showed the co-localization of DAPI (blue), NE (green), and histone H3 (red). Magnification was 100×, and the scale bar length was 20 μm. Arrows indicate extracellular staining of the chosen NET markers.

The NET markers, histone H3 and neutrophil elastase, were detected in 27 and 29 of 33 thrombi, respectively. The presence of NETs in coronary thrombi is illustrated in Figure 2 with co-localization of two NET markers, histone H3 and neutrophil elastase, alongside extracellular DNA.

### 2.3. Investigations in Peripheral Blood

#### 2.3.1. Associations Between PAD4 mRNA, IL-6, IL-6R, and NETs

As for the coronary thrombi, the mRNA of PAD4 and IL-6R mRNA were correlated in circulating leukocytes (Figure 1b). This association remained statistically significant in a multivariate, linear regression model adjusted for age, sex, and symptom duration (Table 3). The serum protein levels of the NET marker H3Cit were correlated with IL-6R protein levels (r*_s_* = 0.40, *p* = 0.02). No other correlations were observed between NET markers and IL-6R protein levels, or between NET markers and IL-6 protein levels.

#### 2.3.2. Comparison of Coronary PAD4 mRNA and Circulating Markers

PAD4 mRNA levels in coronary thrombi correlated with protein levels of PAD4 in peripheral blood measured in serum (r*_s_* = 0.39, *p* = 0.048). PAD4 mRNA levels in thrombi also correlated with dsDNA (r*_s_* = 0.47, *p* = 0.014) measured in plasma, but not with the other NET markers in peripheral blood.

## 3. Discussion

In coronary thrombi from patients with STEMI, we found that PAD4 mRNA levels correlated with IL-6R mRNA expression, as well as myocardial injury measured as troponin T. We also observed correlations between PAD4 mRNA and IL-6R mRNA in circulating leukocytes, and between protein levels of IL-6R and H3Cit in peripheral blood. Together, these findings might suggest that PAD4 links IL-6R signaling and NETosis in STEMI.

Individually, both NETs and IL-6 signaling have been associated with the formation and stabilization of thrombi in myocardial infarction [3,22]. The web-like structure of NETs coated with neutrophil proteins (Figure 2) has prothrombotic and procoagulant effects and hampers fibrinolysis [10,11,23,24,25]. The release of NETs, called NETosis, occurs from minutes to hours after the neutrophil cell is activated, and local NETosis within the coronary thrombi during acute myocardial infarction participates in the prevention of spontaneous reperfusion of the coronary artery [8,26,27,28]. In previous studies, the levels of NETs in coronary thrombi have been correlated with infarct size and clinical outcomes, and our finding that PAD4 mRNA is associated with high levels of troponin T corroborates the importance of NETosis in coronary thrombi for myocardial injury in STEMI [8,14]. Importantly, this is further supported by the correlation between PAD4 mRNA and circulating dsDNA, as both NETosis and myocardial cell damage are potential sources of circulating dsDNA after STEMI. As part of IL-6 signaling, our group has previously shown that the NLRP3-activated pathway, with IL-1β and IL-6 as downstream proteins, is also highly regulated in coronary thrombi, and that local IL-6R mRNA in thrombi is linked to myocardial damage [21]. It has recently been shown that neutrophil cells themselves also release NLRP3 in the early inflammatory phase of myocardial ischemia, and that NLRP3 is not only a precursor but also a stimulus for NETosis [29,30]. Although the IL-6-PAD4 axis has been described in other acute and chronic diseases [31,32], this is, to the best of our knowledge, the first time the interplay between NETosis and IL-6R signaling in coronary thrombi has been investigated. Our demonstration of an association between PAD4 mRNA and IL-6R mRNA aligns with our recent finding that IL-6R inhibition reduces the neutrophil cell’s ability to undergo NETosis and marks PAD4 as an emerging target to mitigate local inflammation [6]. The concept of PAD4 inhibition is supported by experimental models that have shown reductions in NET-induced damage in myocardial infarction by PAD4 inhibition [20,33]. Considering the findings from this study, exploring the effects of PAD4 or IL-6R inhibition on coronary thrombus composition is warranted. Such interventions may not only promote thrombus destabilization but also mitigate ischemia–reperfusion injury in patients with STEMI. As ischemia–reperfusion injury is a significant contributing factor to myocardial damage without effective treatment, it represents an ongoing challenge in modern cardiology [34,35,36].

We observed, like the findings in thrombi, an association between PAD mRNA and IL-6R mRNA in circulating leukocytes, where neutrophils represent the predominant subtype. This observation might suggest that the findings in coronary thrombi were derived from neutrophils, although admixture from other inflammatory cells like macrophages cannot be ruled out. Activated circulating neutrophil cells also play an active role during ischemia–reperfusion injury [36]. NETosis might thus play a role in this detrimental process. In this study, peripheral blood samples were collected simultaneously with thrombus aspiration, i.e., as reperfusion started and before the full effect was presumably reached. We have previously published that coronary thrombi and circulating leukocyte mRNA levels of IL-6 and IL-6R did not correlate with the protein levels of IL-6 and IL-6R in peripheral blood [21], suggesting that peak concentrations of mRNA and protein may occur at distinct time points. Nevertheless, we did find a correlation between the protein levels of the NET marker H3Cit and IL-6R in the circulation. H3Cit is the direct product of PAD4 activity, which was upregulated both in coronary thrombi and circulating leukocytes, and is considered as a specific NET marker [37,38].

### Limitations

The sample size in our study is suitable for hypothesis-generating studies but limits subgroup analysis and increases the risk of type II statistical errors. We only included patients that underwent thrombus aspiration at the discretion of the PCI-operator. These patients might represent a subgroup with higher propensity for thrombus formation compared to the general STEMI population, and their thrombi composition might differ from that of most other patients with STEMI. In addition, the low female study participation rate of 9% limits the generalizability of our results. Moreover, the chosen marker of NETs can be discussed, as NET composition might vary depending on the initial stimuli [39,40], and more ideal markers for STEMI-related research cannot be ruled out. Also, IL-6R exists in two forms, as membrane-bound (mIL-6R) and soluble (sIL-6R). In this study, only sIL-6R was measured in the circulation. In addition, quantification of the immunofluorescence-stained factors in coronary thrombi would have added valuable insight; however, we lacked the necessary expertise and facilities to perform it at the time of the study. Furthermore, the timing of blood sampling may be relevant to our findings. All samples were obtained at the catheterization lab shortly after hospital admission, but the ischemic period ranged from 60 to 1440 min. The processes of translation from mRNA to protein, as well as the elimination of proteins, could be in different stages. Lastly, this is a cross-sectional study, and causality cannot be determined. Future studies are needed to establish the value of these markers in the context of myocardial infarction.

## 4. Materials and Methods

### 4.1. Study Design

The material analyzed in this study was collected for the Thrombus Aspiration in ST-elevation myocardial Infarction (TASTI) trial (registered at clinicaltrials.gov, NCT02746822), as previously described [21]. Briefly, 33 patients with STEMI treated with percutaneous coronary intervention (PCI) and thrombus aspiration were consecutively included between August 2015 and January 2019. Thrombi and peripheral blood samples were obtained simultaneously in the catheterization lab, and an additional peripheral blood sample was drawn the following morning.

### 4.2. Thrombi Preparation

Intracoronary thrombi were obtained using a standard aspiration catheter, rinsed with saline, and divided into two equal portions. One portion was preserved in 10% buffered formalin, processed chemically, and embedded in paraffin for subsequent histological and immunofluorescence analyses. The second portion was snap-frozen in RNA-later solution (Qiagen, Hilden, Germany) and stored at −80 °C to facilitate later RNA extraction and gene expression analysis.

The formalin-fixed, paraffin-embedded (FFPE) thrombi were serially sectioned at 3.5 μm. Sections 1 and 7 were stained with hematoxylin and eosin (HE). For immunofluorescence analysis, the FFPE thrombi were immunofluorescence-stained for histone H3 and neutrophil elastase (Supplementary Tables S1 and S2). Double immunofluorescence was performed sequentially using a Ventana Discovery Ultra automated slide stainer (Ventana Medical System, 750–601, Roche, Basel, Switzerland). Monoclonal mouse anti-human neutrophil elastase (M752, Dako, Glostrup, Denmark) diluted 1:50 in Discovery Antibody Diluent (05266319001, Roche Diagnostics, Basel, Switzerland) was incubated for 32 min, followed by incubation with OmniMap anti-mouse HRP (5269652001, Roche) for 12 min and Discovery FITC (07259212001, Roche Diagnostics, Basel, Switzerland) for 12 min.

Monoclonal rabbit anti-histone H3 (4499S, Cell Signaling Technology, Inc., Danvers, MA, USA) diluted 1:400 in Discovery Antibody Diluent (05266319001, Roche Diagnostics, Basel, Switzerland) was incubated for 32 min, followed by OmniMap anti-Rabbit HRP (05269679001, Roche Diagnostics, Basel, Switzerland) for 12 min and Discovery Rhodamine (07259883001, Roche Diagnostics, Basel, Switzerland) for 12 min. Heat-induced epitope retrieval (HIER) was performed before the second cycle using Discovery CC1 (06414575001, Roche Diagnostics, Basel, Switzerland) for 48 min at 95 °C, and peroxidase was inactivated using a Discovery inhibitor (7017944001, Roche Diagnostics, Basel, Switzerland). Cell nuclei were stained with Discovery QD 4′,6-Diamidino-2-Phenylindole (DAPI) (5268826001, Roche Diagnostics, Basel, Switzerland) for 8 min, and the sections were mounted with ProLong Glass Antifade Mountant (Molecular Probes, Thermo Fisher Scientific, Waltham, MA, USA).

### 4.3. Blood Sampling Protocol

Peripheral venous blood was drawn into tubes without additives for serum analysis and tubes containing EDTA for plasma analysis. Tubes without additives were kept at room temperature for 30–60 min to ensure complete coagulation, followed by centrifugation at 2100× *g* for 10 min at room temperature. EDTA tubes were immediately placed on ice and centrifuged within 30 min at 2500 g for 20 min at 4 °C to procure platelet-poor plasma. Following centrifugation, serum and plasma samples were divided into aliquots and stored at −80 °C for subsequent analyses. BD PAXgene™ Blood RNA tubes were maintained at room temperature for 2–72 h to stabilize leukocyte RNA prior to storage at −80 °C.

### 4.4. Laboratory Analysis

For mRNA quantification, we extracted RNA from the snap-frozen thrombi samples utilizing the High Pure RNA Tissue Kit (Roche Diagnostics GmbH, Mannheim, Germany), supplemented with Proteinase K Solution and stabilized with lysing buffer. Homogenization was achieved using a Thermomixer (Eppendorf AG, Hamburg, Germany) and stainless steel grinding balls (Qiagen GmbH, Hilden, Germany). We isolated RNA from PAXgene tubes with the PAXgene^®^ Blood RNA Kit (PreAnalytix, Qiagen GmbH) and further performed purification using RNeasy^®^ MinElute^®^ Cleanup Kit (Qiagen). RNA quality and concentration (ng/μL) were assessed with the NanoDrop™ 1000 Spectrophotometer (Thermo Scientific, Wilmington, DE, USA). Complementary DNA was synthesized from equal amounts of RNA using the qScript™ cDNA SuperMix (Quanta Biosciences, Gaithersburg, MD, USA). Real-time PCR was conducted on a ViiA™ 7 system (Applied Biosystems, Foster City, CA, USA) with TaqMan^®^ Universal PCR Master Mix (P/N 4324018) and TaqMan^®^ assays for IL-6 (Hs00174131_m1), IL-6R (Hs01075664_m1), and PAD 4 (Hs01057483_m1).

In peripheral blood drawn at admission, dsDNA was quantified in EDTA plasma using the fluorescent nucleic acid stain Quant-iT PicoGreen (Invitrogen, Paisley, UK) and measured by fluorometry (Fluoroskan Ascent, Thermo Fisher Scientific Oy, Vantaa, Finland). Myeloperoxidase–DNA (MPO-DNA) complexes were assessed in undiluted EDTA plasma using an enzyme-linked immunosorbent assay (ELISA) technique previously described by Kessenbrock et al. [41]. Briefly, microplates were coated with the capture antibody anti-MPO (Bio-Rad, Hercules, CA, USA) and incubated overnight at 4 °C. After blocking with bovine serum albumin, patient samples and a peroxidase-labeled anti-DNA antibody (Cell Death Detection kit, Roche Diagnostics GmbH, Mannheim, Germany) were added and incubated for two hours. Following incubation, a peroxidase substrate was introduced, and absorbance was measured and expressed as optical density (OD) units. H3Cit levels were analyzed in serum in a 1:2 dilution with ELISA buffer using a commercial sandwich ELISA kit (Cayman Chemical, Ann Arbor, MI, USA). PAD4, IL-6, and IL-6R were quantified in serum using commercially available ELISA kits (PAD4 (human), Cayman Chemical, Ann arbor, MI, USA and Quantikine^®^ HS ELISA, R&D Systems^®^, Minneapolis, MN, USA). All samples were analyzed on one plate of the specific assays. The intra-assay coefficients of variability were 5.8% (dsDNA), 7.9% (MPO-DNA), 13.2% (H3Cit), 1.4% (PAD4), 10.6% (IL-6), and 3.6% (IL-6R).

Cardiac troponin T was measured in serum collected at admission and on the following day with commercial electrochemiluminescence immunoassay (third-generation cTnT, Elecys 2010, Roche, Mannheim, Germany). The inter-assay coefficient of variability was 7%.

### 4.5. Statistical Methods

The demographic data were given as median (25% and 75% percentiles), mean (SD), or numbers (%) as appropriate. All variables were evaluated for normality by histograms and qq-plots. Correlation analyses were performed by Spearman’s Rho. Linear associations were analyzed with linear regression. Multivariable models were adjusted for the covariates: age, sex, and symptom duration. We chose not to adjust for inflammatory-linked variables like CRP and troponin T. The level of statistical significance was set to two-sided *p* ≤ 0.05. All statistical analyses were performed on STATA v.18 SE (StataCorp LLC, College Station, TX, USA).

## 5. Conclusions

We demonstrate an association between PAD4, IL-6R, and troponin release in STEMI patients. This indicates a PAD4-mediated link between IL-6R and NET formation and highlights PAD4 as a potential treatment target for mitigating inflammation and myocardial injury in STEMI. Further mechanistic and clinical studies are needed to confirm a causal relationship and clarify the therapeutic potential of PAD4 inhibition in STEMI.

## Figures and Tables

**Table 1:** Baseline characteristics.

	Total *n* = 33
Age, years	60 (±11)
Females	3 (9%)
Current or previous smoker	27 (84%)
BMI, kg/m^2^	27.7 (23.4, 28.6)
sBP, mmHg	127 (±31)
dBP, mmHg	82 (±19)
Heart rate	70 (65, 90)
Prior myocardial infarction	1 (3%)
Prior hypertension	11 (33%)
Prior diabetes mellitus type 2	4 (12%)
Prior medication:	
Acetylsalicylic acid	6 (18%)
P2Y12-inhibitor	2 (6%)
Anticoagulation	3 (9%)
RAAS inhibitors	5 (15%)
Betablocker	4 (12%)
Aldosterone antagonist	0 (0%)
Statins	6 (18%)
Symptom start to PCI, min	152 (122, 343)
Culprit artery:	
LAD	16 (48%)
Cx	6 (18%)
RCA	11 (33%)
Troponin T peak, µg/L	3434 (1250, 6967)
CRP, mg/L	2.71 (0.96, 6.03)

Values are given as mean (±SD), median (IQR 25, 75), or proportions (%). BMI = body mass index, sBP = systolic blood pressure, dPB = diastolic blood pressure, RAAS = renin–angiotensin–aldosterone system, PCI = percutaneous coronary intervention, LAD = left anterior descending artery, Cx = circumflex artery, RCA = right coronary artery, CRP = C-reactive protein.

**Table 2:** Available markers and their locations.

	Gene Expression in Thrombi	Gene Expression in Circulating Leukocytes	Circulating Levels
IL-6	√	√	√
IL-6R	√	√	√
PAD4	√	√	√
dsDNA	x	x	√
MPO-DNA	x	x	√
H3Cit	x	x	√

All circulating proteins were quantified by ELISA in serum, except MPO-DNA, which was quantified in EDTA plasma. dsDNA was stained with PicoGreen in EDTA plasma and quantified with fluorometry. IL-6 = intereleukin-6, IL-6R = interleukin-6 receptor, PAD4 = peptidylarginine deiminase 4, dsDNA = double-stranded DNA, MPO-DNA = myeloperioxidase–DNA, H3Cit = citrullinated histone H3.

**Table 3:** Associations between PAD4 mRNA, IL-6R mRNA, and troponin T in thrombi and PAD4 mRNA and IL-6R mRNA in leukocytes.

	Univariate			Multivariate		
	β	CI	*p*-value	β	CI	*p*-value
Association with PAD4 mRNA in coronary thrombi						
IL-6R mRNA	0.34	0.16–0.51	0.001	0.34	0.16–0.53	0.001 *
Circulating peak troponin T, quartile q4 vs. 1–3	1.0	0.20–1.80	0.016	1.15	0.27–2.04	0.013 *
Association with PAD4 mRNA in circulating leukocytes						
IL-6R mRNA	0.98	0.18–1.77	0.017	0.92	0.07–1.77	0.036 *

Univariate and multivariate linear regression models of associations between mRNA in coronary thrombi (orange) and circulating leukocytes (green). mRNA levels were measured by qPCR. PAD4 = peptidylarginine deiminase 4, IL-6R = interleukin-6 receptor. * Adjusted for age, sex, and symptom duration.

## Data Availability

Data will be available upon reasonable request.

## Supplementary Materials

The following supporting information can be downloaded at https://www.mdpi.com/article/10.3390/ijms26115348/s1.

## Abbreviations

The following abbreviations are used in this manuscript:

STEMI	ST-elevation myocardial infarction
Il-6	Interleukin-6
IL-6R	Interleukin-6 receptor
NETs	Neutrophil extracellular traps
PAD4	Peptidylarginine deiminase 4
dsDNA	Double-stranded deoxyribonucleic acid
H3Cit	Citrullinated histone H3
MPO-DNA	Myeloperioxidase bound to deoxyribonucleic acid
NLRP3	NOD-, LRR-, and pyrin domain-containing protein 3
ASSAIL-MI	Assessing the effect of anti-IL-6 treatment in myocardial infarction
PCI	Percutaneous coronary intervention
FFPE	Formalin-fixed, paraffin-embedded
ELISA	Enzyme-linked immunosorbent assay

## References

[1] Tollefsen I.M., Shetelig C., Seljeflot I., Eritsland J., Hoffmann P., Andersen G.O. (2021). High levels of interleukin-6 are associated with final infarct size and adverse clinical events in patients with STEMI. Open Heart.

[2] Groot H.E., Al Ali L., van der Horst I.C.C., Schurer R.A.J., van der Werf H.W., Lipsic E., van Veldhuisen D.J., Karper J.C., van der Harst P. (2019). Plasma interleukin 6 levels are associated with cardiac function after ST-elevation myocardial infarction. Clin. Res. Cardiol..

[3] Libby P. (2021). Targeting Inflammatory Pathways in Cardiovascular Disease: The Inflammasome, Interleukin-1, Interleukin-6 and Beyond. Cells.

[4] Broch K., Anstensrud A.K., Woxholt S., Sharma K., Tollefsen I.M., Bendz B., Aakhus S., Ueland T., Amundsen B.H., Damas J.K. (2021). Randomized Trial of Interleukin-6 Receptor Inhibition in Patients With Acute ST-Segment Elevation Myocardial Infarction. J. Am. Coll. Cardiol..

[5] Feng Y., Ye D., Wang Z., Pan H., Lu X., Wang M., Xu Y., Yu J., Zhang J., Zhao M. (2022). The Role of Interleukin-6 Family Members in Cardiovascular Diseases. Front. Cardiovasc. Med..

[6] Kindberg K.M., Broch K., Andersen G.O., Anstensrud A.K., Akra S., Woxholt S., Tollefsen I.M., Ueland T., Amundsen B.H., Klow N.E. (2024). Neutrophil Extracellular Traps in ST-Segment Elevation Myocardial Infarction: Reduced by Tocilizumab and Associated With Infarct Size. JACC Adv..

[7] Brinkmann V., Reichard U., Goosmann C., Fauler B., Uhlemann Y., Weiss D.S., Weinrauch Y., Zychlinsky A. (2004). Neutrophil extracellular traps kill bacteria. Science.

[8] Mangold A., Alias S., Scherz T., Hofbauer M., Jakowitsch J., Panzenbock A., Simon D., Laimer D., Bangert C., Kammerlander A. (2015). Coronary neutrophil extracellular trap burden and deoxyribonuclease activity in ST-elevation acute coronary syndrome are predictors of ST-segment resolution and infarct size. Circ. Res..

[9] Doring Y., Soehnlein O., Weber C. (2017). Neutrophil Extracellular Traps in Atherosclerosis and Atherothrombosis. Circ. Res..

[10] Nappi F., Bellomo F., Avtaar Singh S.S. (2023). Worsening Thrombotic Complication of Atherosclerotic Plaques Due to Neutrophils Extracellular Traps: A Systematic Review. Biomedicines.

[11] Varju I., Longstaff C., Szabo L., Farkas A.Z., Varga-Szabo V.J., Tanka-Salamon A., Machovich R., Kolev K. (2015). DNA, histones and neutrophil extracellular traps exert anti-fibrinolytic effects in a plasma environment. Thromb. Haemost..

[12] Folco E.J., Mawson T.L., Vromman A., Bernardes-Souza B., Franck G., Persson O., Nakamura M., Newton G., Luscinskas F.W., Libby P. (2018). Neutrophil Extracellular Traps Induce Endothelial Cell Activation and Tissue Factor Production Through Interleukin-1alpha and *Cathepsin G*. Arterioscler. Thromb. Vasc. Biol..

[13] Helseth R., Shetelig C., Andersen G.O., Langseth M.S., Limalanathan S., Opstad T.B., Arnesen H., Hoffmann P., Eritsland J., Seljeflot I. (2019). Neutrophil Extracellular Trap Components Associate with Infarct Size, Ventricular Function, and Clinical Outcome in STEMI. Mediat. Inflamm..

[14] Blasco A., Coronado M.J., Vela P., Martin P., Solano J., Ramil E., Mesquida A., Santos A., Cozar B., Royuela A. (2022). Prognostic Implications of Neutrophil Extracellular Traps in Coronary Thrombi of Patients with ST-Elevation Myocardial Infarction. Thromb. Haemost..

[15] Novotny J., Chandraratne S., Weinberger T., Philippi V., Stark K., Ehrlich A., Pircher J., Konrad I., Oberdieck P., Titova A. (2018). Histological comparison of arterial thrombi in mice and men and the influence of Cl-amidine on thrombus formation. PLoS ONE.

[16] Riegger J., Byrne R.A., Joner M., Chandraratne S., Gershlick A.H., Ten Berg J.M., Adriaenssens T., Guagliumi G., Godschalk T.C., Neumann F.J. (2016). Histopathological evaluation of thrombus in patients presenting with stent thrombosis. A multicenter European study: A report of the prevention of late stent thrombosis by an interdisciplinary global European effort consortium. Eur. Heart J..

[17] Fuchs T.A., Brill A., Duerschmied D., Schatzberg D., Monestier M., Myers D.D., Wrobleski S.K., Wakefield T.W., Hartwig J.H., Wagner D.D. (2010). Extracellular DNA traps promote thrombosis. Proc. Natl. Acad. Sci. USA.

[18] Gould T.J., Vu T.T., Swystun L.L., Dwivedi D.J., Mai S.H., Weitz J.I., Liaw P.C. (2014). Neutrophil extracellular traps promote thrombin generation through platelet-dependent and platelet-independent mechanisms. Arter. Thromb. Vasc. Biol..

[19] Thiam H.R., Wong S.L., Qiu R., Kittisopikul M., Vahabikashi A., Goldman A.E., Goldman R.D., Wagner D.D., Waterman C.M. (2020). NETosis proceeds by cytoskeleton and endomembrane disassembly and PAD4-mediated chromatin decondensation and nuclear envelope rupture. Proc. Natl. Acad. Sci. USA.

[20] Du M., Yang W., Schmull S., Gu J., Xue S. (2020). Inhibition of peptidyl arginine deiminase-4 protects against myocardial infarction induced cardiac dysfunction. Int. Immunopharmacol..

[21] Nordeng J., Schandiz H., Solheim S., Akra S., Hoffman P., Roald B., Bendz B., Arnesen H., Helseth R., Seljeflot I. (2021). The Inflammasome Signaling Pathway Is Actively Regulated and Related to Myocardial Damage in Coronary Thrombi from Patients with STEMI. Mediat. Inflamm..

[22] Thalin C., Hisada Y., Lundstrom S., Mackman N., Wallen H. (2019). Neutrophil Extracellular Traps: Villains and Targets in Arterial, Venous, and Cancer-Associated Thrombosis. Arter. Thromb. Vasc. Biol..

[23] Bonaventura A., Vecchie A., Abbate A., Montecucco F. (2020). Neutrophil Extracellular Traps and Cardiovascular Diseases: An Update. Cells.

[24] Farkas A.Z., Farkas V.J., Gubucz I., Szabo L., Balint K., Tenekedjiev K., Nagy A.I., Sotonyi P., Hidi L., Nagy Z. (2019). Neutrophil extracellular traps in thrombi retrieved during interventional treatment of ischemic arterial diseases. Thromb. Res..

[25] Longstaff C., Varju I., Sotonyi P., Szabo L., Krumrey M., Hoell A., Bota A., Varga Z., Komorowicz E., Kolev K. (2013). Mechanical stability and fibrinolytic resistance of clots containing fibrin, DNA, and histones. J. Biol. Chem..

[26] Yipp B.G., Kubes P. (2013). NETosis: How vital is it?. Blood.

[27] McGill C.J., Lu R.J., Benayoun B.A. (2021). Protocol for analysis of mouse neutrophil NETosis by flow cytometry. STAR Protoc..

[28] Stakos D.A., Kambas K., Konstantinidis T., Mitroulis I., Apostolidou E., Arelaki S., Tsironidou V., Giatromanolaki A., Skendros P., Konstantinides S. (2015). Expression of functional tissue factor by neutrophil extracellular traps in culprit artery of acute myocardial infarction. Eur. Heart J..

[29] Heger L.A., Schommer N., Van Bruggen S., Sheehy C.E., Chan W., Wagner D.D. (2024). Neutrophil NLRP3 promotes cardiac injury following acute myocardial infarction through IL-1beta production, VWF release and NET deposition in the myocardium. Sci. Rep..

[30] Munzer P., Negro R., Fukui S., di Meglio L., Aymonnier K., Chu L., Cherpokova D., Gutch S., Sorvillo N., Shi L. (2021). NLRP3 Inflammasome Assembly in Neutrophils Is Supported by PAD4 and Promotes NETosis Under Sterile Conditions. Front. Immunol..

[31] Yahagi A., Saika T., Hirano H., Takai-Imamura M., Tsuji F., Aono H., Iseki M., Morita Y., Igarashi H., Saeki Y. (2019). IL-6-PAD4 axis in the earliest phase of arthritis in knock-in gp130F759 mice, a model for rheumatoid arthritis. RMD Open.

[32] Kang L., Yu H., Yang X., Zhu Y., Bai X., Wang R., Cao Y., Xu H., Luo H., Lu L. (2020). Neutrophil extracellular traps released by neutrophils impair revascularization and vascular remodeling after stroke. Nat. Commun..

[33] Yang K., Gao R., Chen H., Hu J., Zhang P., Wei X., Shi J., Chen Y., Zhang L., Chen J. (2024). Myocardial reperfusion injury exacerbation due to ALDH2 deficiency is mediated by neutrophil extracellular traps and prevented by leukotriene C4 inhibition. Eur. Heart J..

[34] Hausenloy D.J., Yellon D.M. (2013). Myocardial ischemia-reperfusion injury: A neglected therapeutic target. J. Clin. Investig..

[35] Yellon D.M., Hausenloy D.J. (2007). Myocardial reperfusion injury. N. Engl. J. Med..

[36] Shah M., Yellon D.M., Davidson S.M. (2020). The Role of Extracellular DNA and Histones in Ischaemia-Reperfusion Injury of the Myocardium. Cardiovasc. Drugs Ther..

[37] Saisorn W., Santiworakul C., Phuengmaung P., Siripen N., Rianthavorn P., Leelahavanichkul A. (2024). Extracellular traps in peripheral blood mononuclear cell fraction in childhood-onset systemic lupus erythematosus. Sci. Rep..

[38] Paues Goranson S., Thalin C., Lundstrom A., Hallstrom L., Lasselin J., Wallen H., Soop A., Mobarrez F. (2018). Circulating H3Cit is elevated in a human model of endotoxemia and can be detected bound to microvesicles. Sci. Rep..

[39] Chen T., Li Y., Sun R., Hu H., Liu Y., Herrmann M., Zhao Y., Munoz L.E. (2021). Receptor-Mediated NETosis on Neutrophils. Front. Immunol..

[40] Petretto A., Bruschi M., Pratesi F., Croia C., Candiano G., Ghiggeri G., Migliorini P. (2019). Neutrophil extracellular traps (NET) induced by different stimuli: A comparative proteomic analysis. PLoS ONE.

[41] Kessenbrock K., Krumbholz M., Schonermarck U., Back W., Gross W.L., Werb Z., Grone H.J., Brinkmann V., Jenne D.E. (2009). Netting neutrophils in autoimmune small-vessel vasculitis. Nat. Med..

