# Supplementary material for: IL-6R Signaling Is Associated with PAD4 and Neutrophil Extracellular Trap Formation in Patients with STEMI
Source: Int J Mol Sci. 2025 Jun 2;26(11):5348. doi: 10.3390/ijms26115348 (PMC12154504; doi:10.3390/ijms26115348)
Supplement: Supplementary file 1 [file ijms-26-05348-s001.zip › ijms-3620157-supplementary.pdf]

Supplementary Materials

Table S1. Localization by immunofluorescence staining.

Immune marker	Cell type	Localization
Histone H3	Monocytes and neutrophils	Nuclear, cytoplasmic, membranous and extracellular
Neutrophil Elastase	Neutrophils	Cytoplasmic, membranous, and extracellular

Cell types, and localization within the cells, of the different markers visualized with immunofluorescence staining.

Table S2. Details about the chosen antibodies.

Antibody	Source	Dilution
Histone H3	4499S, Cell Signaling<br>Monoclonal Rabbit	1/400
Neutrophil Elastase	M752, Dako<br>Monoclonal Mouse	1/50
